# Viscount Knutsford's Reply in *the Times*

**Published:** 1918-07-13

**Authors:** 

**Affiliations:** Chairman, London Hospital. London Hospital, Whitechapel, E. 1


### LONDON HOSPITAL NURSES.

### Viscount Knutsford's Reply in "The Times."

Sir,—That "honourable and gallant" member for Stirlingshire, Major Dr. Chapple, asked a question in the House of Commons on Thursday last. It is an old trick, this asking a question and giving a stab, and he got a well-earned rebuke from the Speaker. Under the protection of a question, Dr. Chapple suggested that "the London Hospital sends out nurses, who are not fully trained, as private nurses to outside patients, and 'farms out' its nurses, paying them 13s. a week, and charging the engager two guineas a week for the nurses' services." Both statements are untrue.

The London Hospital has never sent out private nurses who are not fully trained. The time necessary to train a nurse is not a fixed one. It may take two or three or any number of years, according to the facilities obtainable at the hospital giving the training, and, very specially, to the organisation of that training. At the "London" we can train a nurse in two years, and as London Hospital nurses are honoured and appreciated by thousands, from King to cobbler, and hold the highest positions in the nursing world to-day, it would seem that doctors (except Dr. Chapple) and the public consider that the training is good.

As to the charge of "farming out" our nurses. We pay our private staff from £35 to £55 a year and 2s. 6d. a week for washing. The rate of pay is not all, however. After eighteen years from the date of entering she, on leaving, receives a full-pay pension for life, although she may take up any other appointment. She is allowed one month's holiday a year (full pay) and one day off for every fourteen days she is at a case. Between her cases she returns to the hospital as her home. If ill she is nursed in private quarters at the hospital, and receives full pay during her illness. I remember two cases where this was given for over a year. A nurse "on her own" does certainly take the whole of the fee. But she has to pay for her room. She has no pension to look forward to. She has to pay for her holidays, during which she is not earning. She uses up her savings in the gaps between her cases. If ill she is not only not earning, but has to bear the expense of her illness. The charge that we are "farming out" or "sweating" our nurses is not true, therefore. At any rate, the nurses themselves are not of that opinion, or why do they prefer to stay with us instead of going "on their own," as they are free to do? Quite a number of them even stay with us after they are entitled to their pension.—Yours faithfully,

Knutsford, Chairman, London Hospital.

London Hospital, Whitechapel, E. 1,
July 8.

